# Supplementary material for: HDAC Inhibitors Enhance Efficacy of the Oncolytic Adenoviruses Ad∆∆ and Ad-3∆-A20T in Pancreatic and Triple-Negative Breast Cancer Models
Source: Viruses. 2022 May 9;14(5):1006. doi: 10.3390/v14051006 (PMC9143155; doi:10.3390/v14051006)
Supplement: Supplementary file 1 [file viruses-14-01006-s001.zip › viruses-1687352-supplementary.pdf]

**Supplementary Table S1.** Characteristics of the TNBC and PDAC cell lines used in the study.

| | Origin | Subtype | p53 status | Major mutations | Other alterations |
|---|---|---|---|---|---|
| BT549 | TNBC<br>Primary tumour | Infiltrating ductal carcinoma | Mutated | PTEN deleted | Do not express ER, PR, HER2; BRCA1 wt |
| CAL51 | TNBC, Metastatic; pleural effusion | Adenocarcinoma | Wildtype | PTEN silenced | Do not express ER, PR, HER2 |
| HCC1143 | TNBC<br>Primary tumour | Infiltrating ductal carcinoma | Mutant expressed | PTEN not determined | Do not express ER, PR, HER2; BRCA1 wt |
| MDAMB436 | TNBC, Metastatic; pleural effusion | Infiltrating ductal carcinoma | Mutated | Low levels of PTEN expression | Do not express ER, PR, HER2; BRCA1 mutant |
| SUM149 | TNBC<br>Primary tumour | Inflammatory BCa | Mutated | PTEN wildtype | Do not express ER, PR, HER2; BRCA1 mutant |
| SUM159 | TNBC<br>Primary tumour | Infiltrating ductal carcinoma | Mutated | PI3KC mutated | Do not express ER, PR, HER2; BRCA1 wt |
| MiaPaCa2 | PDAC<br>Primary tumour | - | Mutated | KRAS mutated | CDKN2A/p16 deleted; SMAD4 wt |
| Panc04.03 | PDAC<br>Primary tumour | - | Mutated | KRAS mutated | CDKN2A/p16 mutated |
| Suit2 | PDAC<br>Liver metastasis | - | Mutated | KRAS mutated | CDKN2A/p16 silenced |
| PT45 | PDAC<br>Primary tumour | - | Mutated | KRAS mutated | ND |
| BxPC3 | PDAC<br>Primary tumour | - | Mutated | KRAS wt | CDKN2A/p16 wt; SMAD4 deleted |

Data combined from Chavez et al., Breast Dis. 2010;32(1-2);35-48; Deer et al., Pancreas 2010; 39(4);425-435; https://web.expasy.org/cellosaurus/CVCL

**Supplementary Figure S1.**

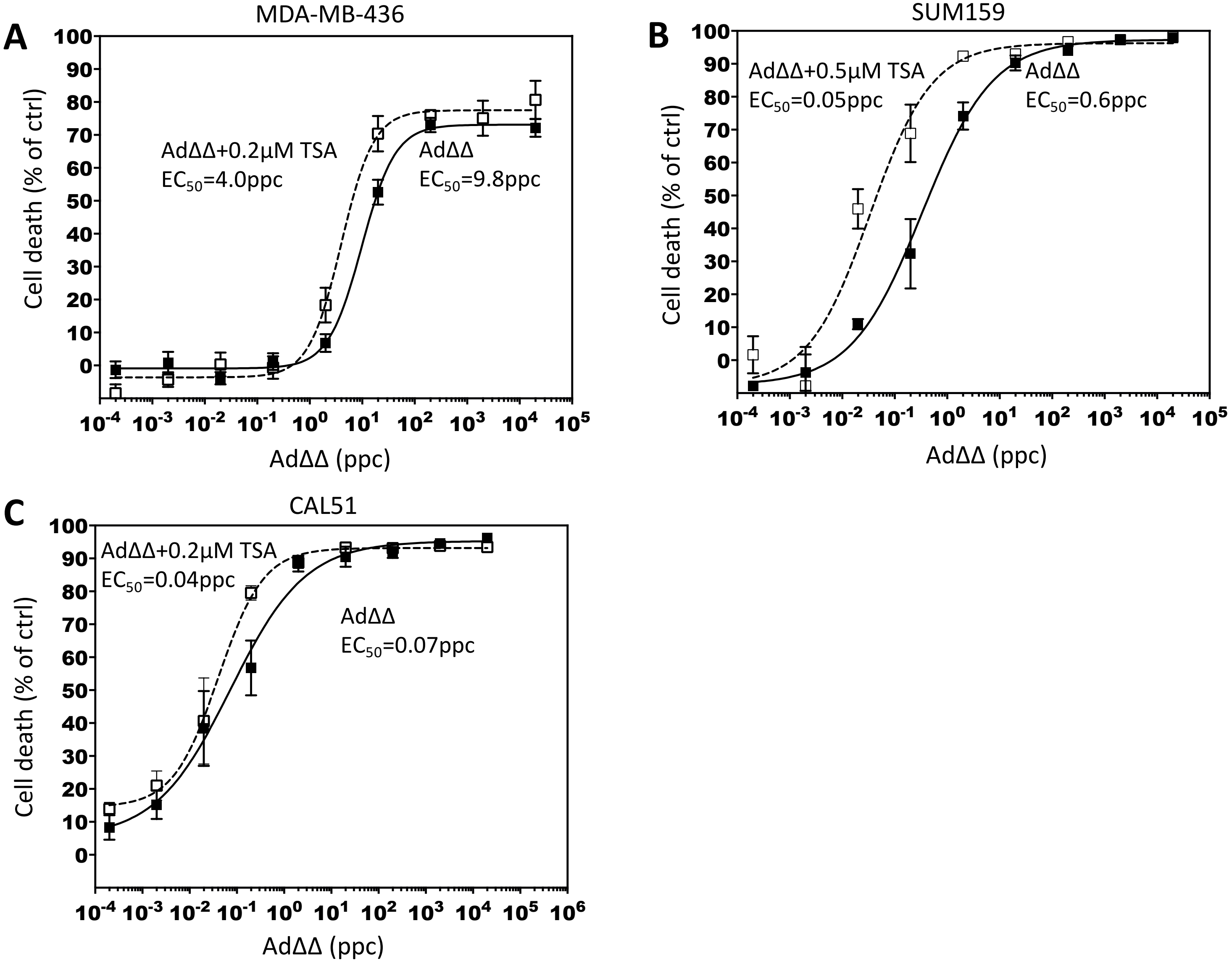


**Supplementary Figure S1. TSA sensitises TNBC cells to AdΔΔ-mediated cell killing.** Dose-response curves to AdΔΔ in MDA-MB-436 **(A)**, SUM159 **(B)** and CAL51 **(C)** cells ±0.2μM TSA (MDA-MB-436 and CAL51) or 0.5μM TSA (SUM159) added simultaneously (=) to infection. Cell death was determined by MTS-viability assays 6d post-infection with 10-fold dilutions of AdΔΔ. One representative study out of three, each in triplicates.

**Supplementary Figure S2.**

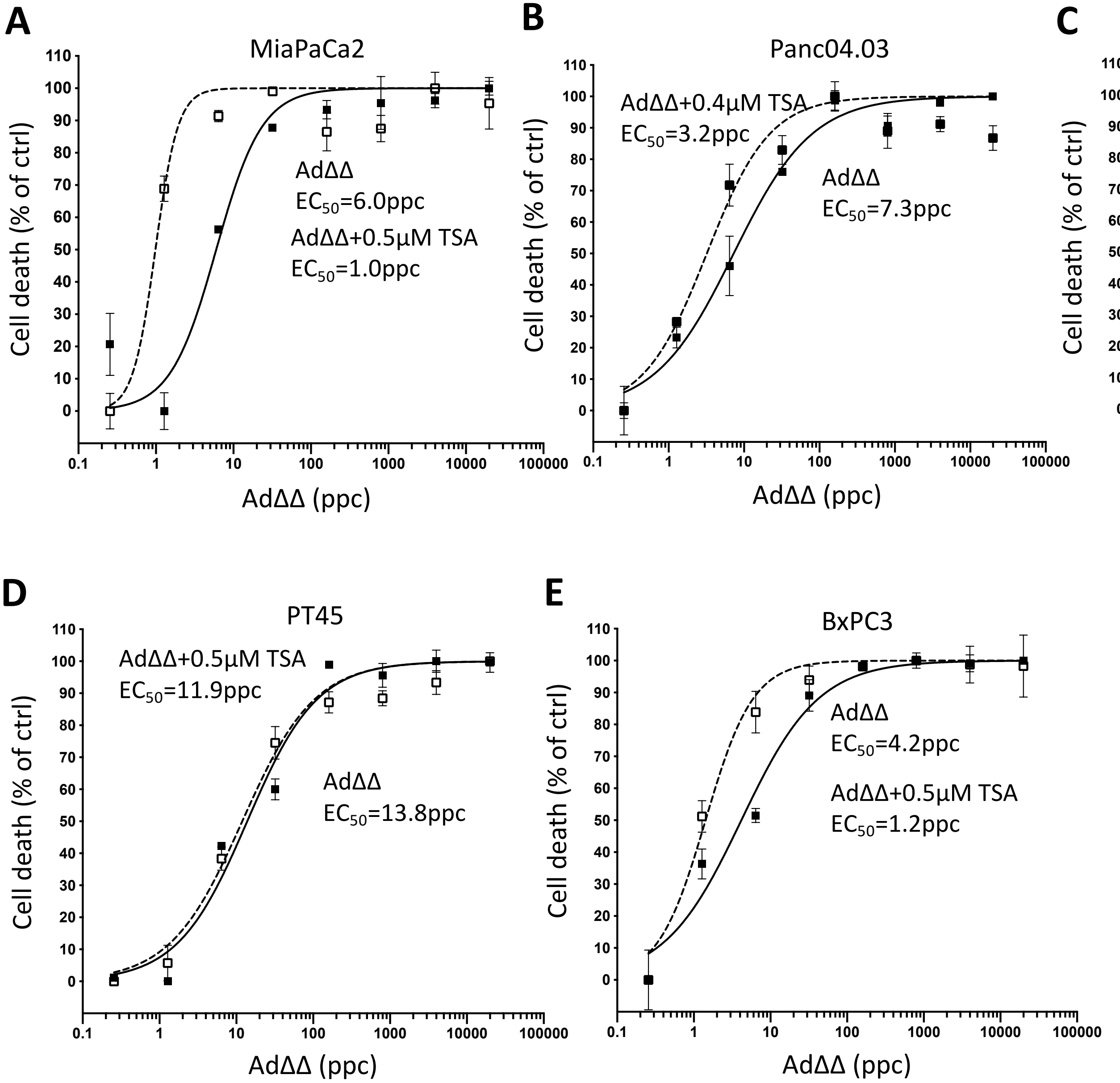


**Supplementary Figure S2. TSA sensitises PDAC cells to AdΔΔ-mediated cell killing.** Dose-response curves to AdΔΔ in MiaPaCA2 **(A)**, Panc04.03 **(B)**, Suit2 **(C)**, PT45 **(D)** and BxPC3 **(E)** cells ± TSA at indicated doses added simultaneously to infection. Cell death was determined by MTS-viability assays 6d post-infection with 5-fold dilutions of AdΔΔ. One representative study out of five, each in duplicates.

**Supplementary Figure S3**

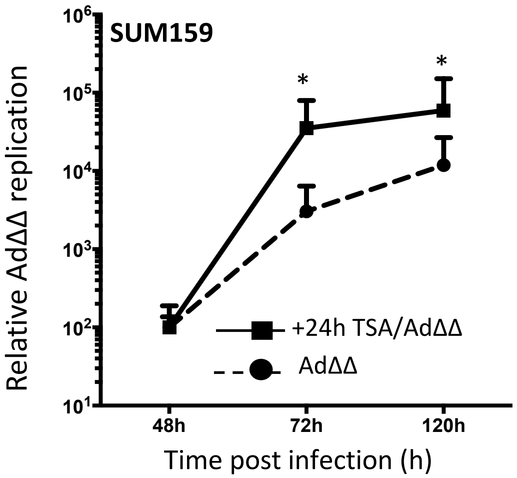


**Supplementary Figure S3 TSA significantly increases the replication rate over time of AdΔΔ (100ppc) when added 24h after infection in SUM159 cells**. Infectious units (pfu/ml) was determined by TCID50 assays and expressed as percentages of the respective treatment at 48h. Data expressed as averages ± SD, *$p<0.05$, n=2.

## Supplementary Figure S4

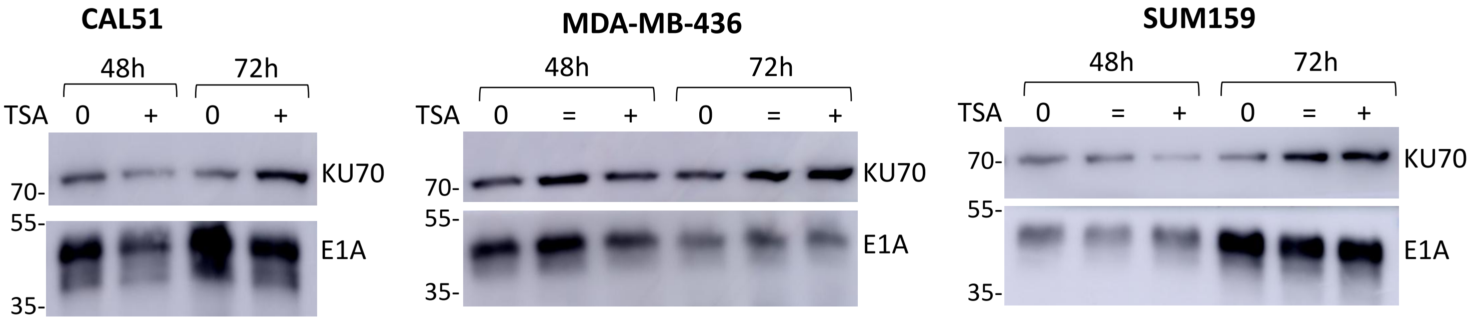


**Supplementary Figure S4. Viral E1A expression in CAL51, MDA-MB-436 and SUM159 cells**. Cells were infected at 10ppc ± 0.25µM TSA added 24h after (+) and at the same time (=) of infection, or with AdΔΔ alone (0). Cells were harvested at the indicated timepoints after infection, lysed and 18.5µg of total protein were loaded/lane. Blots were probed with the respective antibodies and visualized using ECL. Representative blots of >4 experiments.

## Supplementary Figure S5

A

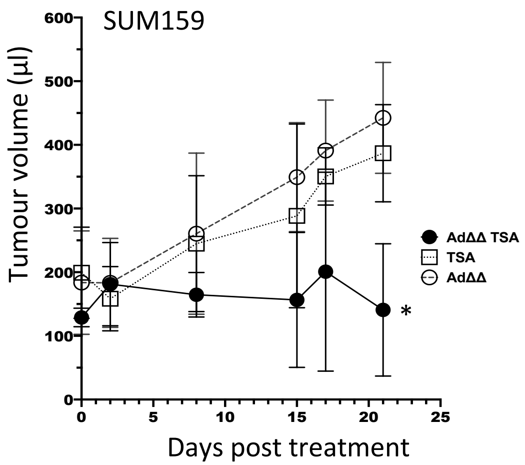


**Supplementary Figure S5 Efficacy of AdΔΔ in combination with TSA in animals with SUM159 and distribution in Suit2 xenografts. A)** SUM159 xenografts in athymic mice treated with AdΔΔ at $1x10^{10}$vp on day 1, 3 and 7 intratumourally or 1.5µg TSA/g on day 2, 4 and 8 intraperitoneally or combined treatment with AdΔΔ+TSA at the same dose schedule. Growth curves shown for day 1 - 21 after last day of treatment, *p<0.05 (AdΔΔ+TSA vs AdΔΔ and TSA alone), 6 animals/group. **B)** Pre-treatment with TSA in animals with Suit2 xenografts support expression of the early viral E1A protein. Animals were administered with 20µg/200µl TSA intraperitoneally on day 1, 4, ad 8 and AdΔΔ ($1x10^9$vp/50µl) injected intratumourally on day 2, 5 and 9. Tumours were harvested 25 days after the final virus administration, fixed and stained for E1A expression. Representative images, 3 animals/group.

B

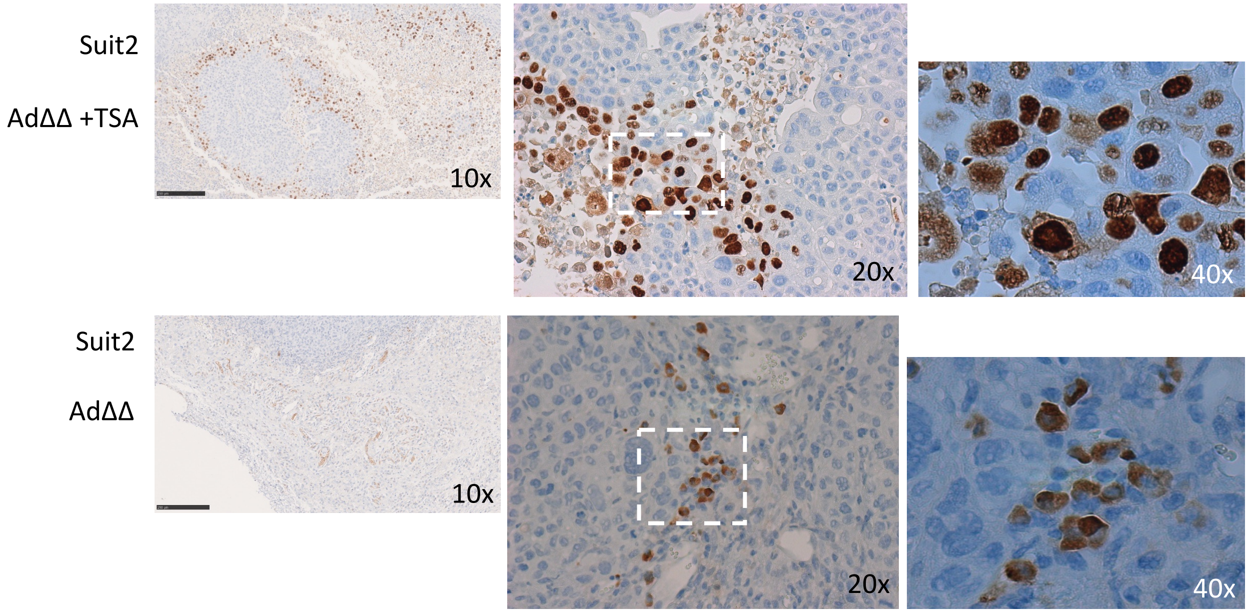

# Supplementary Table S2

**Supplementary Table S2.** Scriptaid sensitises TNBC cells to AdΔΔ mediated cell killing.

| | AdΔΔ | Scriptaid | Scriptaid | Scriptaid |
|---|---|---|---|---|
| **CAL51** | | *0.25µM (=)* | *0.25µM (+)* | *0.5µM (=)* |
| | 0.81ppc | 0.60ppc | 0.98ppc | 0.59ppc |
| **MDA436** | | *0.25µM (=)* | *0.25µM (+)* | *0.5µM (=)* |
| | 42.4ppc | 13.9ppc | 16.0ppc | 14.2ppc |
| **SUM159** | | *0.5µM (=)* | *0.5µM (+)* | *0.25µM (=)* |
| | 7.2ppc | 1.7ppc | 2.7ppc | 1.2ppc |

$EC_{50}$ values for AdΔΔ and Scriptaid (0.25 and 0.5µM) added simultaneously (=) or 24h post infection (+) in MDA-MB-436, SUM159 and CAL51 cells determined by dose-response curves analysed 6d post-infection. One representative study out of three, each in triplicates

**Supplementary Figure S6.**

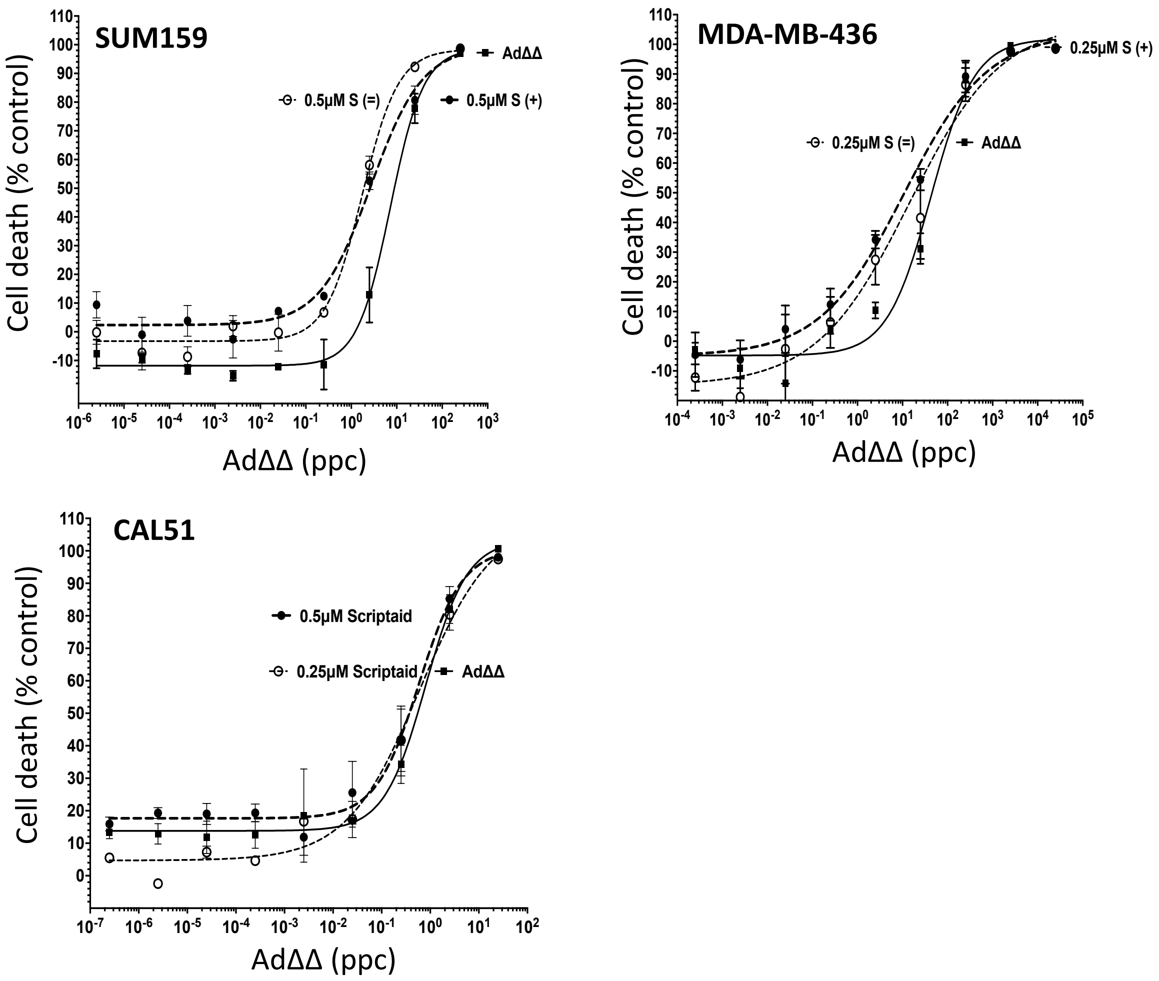


**Supplementary Figure S6. Addition of Scriptaid sensitises SUM159, MDA-MB-436 and CAL51 cells to AdΔΔ.** Dose-response curve to AdΔΔ ± Scriptaid (0.5 and 0.25µM) added simultaneously (=) and after (+) virus. Cell death was determined by MTS-viability assays 6d post-infection. One representative study out of three, each in triplicates.

## Supplementary Figure S7.

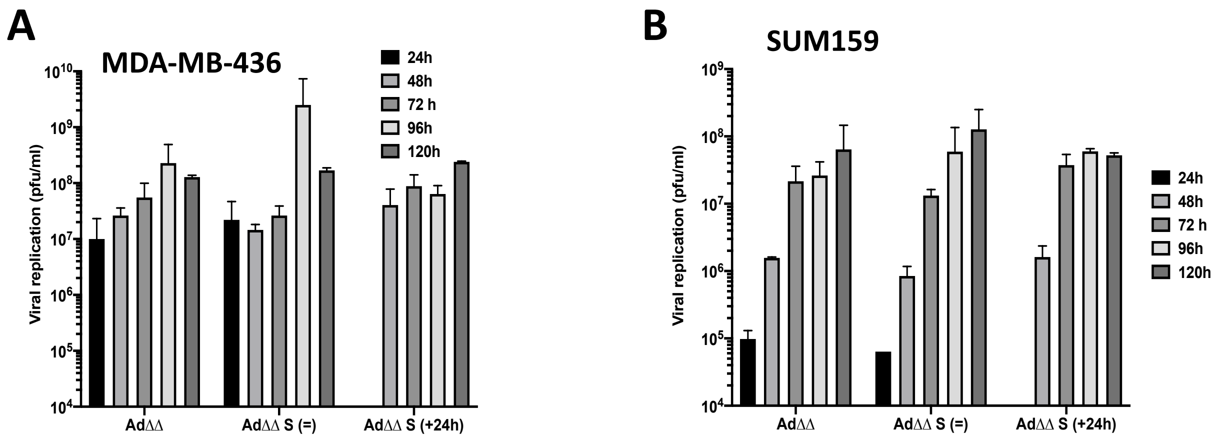


**Supplementary Figure S7. Addition of Scriptaid supports viral replication in TNBC cell lines**. **A-B)** MDA-MB-436 and SUM159 cells were infected with AdΔΔ at 100ppc and treated with Scriptaid at 0.25µM (MDA-MB-436) or 0.5µM (SUM159), added simultaneously (=) or 24h (+) after AdΔΔ infection. Cells and medium were harvested at the indicted time (24-120h) post-infection. Total viral replication (pfu/ml) in each sample was determined as cytopathic effect (CPE) 10d post-infection on JH293 detector cells. Data are expressed as averages ± SD (n=2, each in duplicates).

# Supplementary Figure S8

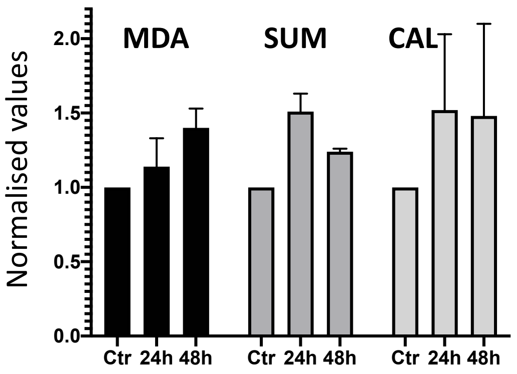


**Supplementary Figure S8. TNBC cell lines express the cancer-specific αvß6-integrin**. Quantification of immunblot shown in Figure 4B Detection of αvß6-integrin (85kDa) and PCNA (29kDa), post-addition of 0.25µM Scriptaid (CAL51) or 0.5µM Scriptaid (MDA-MB-436 and SUM159). Cells were harvested 24h and 48h after treatment and processed for SDS-PAGE and immmunoblotting. Bands were quantified using Image J and ratio of integrin band density to PCNA was normalised to untreated cells, averages of 4 blots from 2 biological repeats.
